# Applying MAPPs Assays to Assess Drug Immunogenicity

**DOI:** 10.3389/fimmu.2020.00698

**Published:** 2020-04-21

**Authors:** Anette C. Karle

**Affiliations:** Novartis Institute for Biomedical Research, Novartis Pharma AG, Basel, Switzerland

**Keywords:** MAPPs, immunogenicity, HLA, proteomics, dendritic cells, drug development, ABIRISK, biotherapeutics

## Abstract

Immunogenicity against biotherapeutic proteins (BPs) and the potential outcome for the patient are difficult to predict. *In vitro* assays that can help to assess the immunogenic potential of BPs are not yet used routinely during drug development. MAPPs (MHC-associated peptide proteomics) is one of the assays best characterized regarding its value for immunogenicity potential assessment. This review is focusing on recent studies that have employed human HLA class II-MAPPs assays to rank biotherapeutic candidates, investigate clinical immunogenicity, and understand mechanistic root causes of immunogenicity. Advantages and challenges of the technology are discussed as well as the different areas of application.

## Introduction

Anti-drug antibodies (ADAs) have been shown to have broad and diverse effects ranging from any clinical significance to loss of efficacy of the BP, impact on PK/PD profile, hypersensitivity responses, or in the worst case the cross-reactivity and neutralization of the endogenous counterpart of the BP. Understanding and predicting immunogenicity against BPs is a major challenge. There is a trend in using *in vitro* assays to assess the immunogenic potential of biotherapeutics, but they are not used routinely during drug development. For several years, MAPPs (MHC associated peptide proteomics) has been applied on many different types of immunological questions (Table 1) and can be considered as the assay best characterized regarding its value for immunogenicity potential assessment. While the use of the MAPPs technology for the identification of immunogenic hotspots of biotherapeutics has been reviewed previously (1), this review is focusing on recent studies that have employed human HLA class II-MAPPs assays to rank biotherapeutic candidates, investigate clinical immunogenicity, and understand mechanistic root causes of immunogenicity.

**Table 1:** Summary of studies that employed MAPPs for candidate ranking, investigative, and mechanistic purposes.

**References**	**MAPPs application**	**Purpose**
Karle et al. (6)	Marketed monoclonal antibodies were compared regarding the number of naturally presented sequence regions.	Ranking of candidates
Xue et al. (13)	Determination of naturally presented sequences of an anti-IL-21R blocking antibody ATR-107.	Investigative
Lamberth et al. (14)	Determination of naturally presented peptides of vatreptacog alfa, a bioengineered analog of recombinant FVIIa.	Investigative
Hamze et al. (8)	Determination of naturally presented sequences of infliximab and rituximab from healthy donors. Determination of T cell epitopes in healthy drug-naïve donors as well as patients treated with infliximab or rituximab.	Investigative
Sekiguchi et al. (7)	Determination of naturally presented sequences of infliximab and adalimumab.	Investigative
Cassotta et al. (10)	Natalizumab-derived HLA-DR associated peptides identified from natalizumab-specific EBV-B clones generated from patients that had developed immunogenicity against natalizumab.	Investigative
Jankowski et al. (12)	MAPPs applied to investigate differences in peptide presentation between different FVIII therapeutics.	Investigative
This study	MAPPs applied to determine naturally presented peptides of human FVIII.	Investigative
Spindeldreher et al. (11)	MAPPs-assisted T cell epitope determination of ixekizumab.	Investigative
Meunier et al. (9)	Determination of naturally presented sequences and T cell epitopes of adalimumab and natalizumab in healthy donors.	Investigative
Karle et al. (15)	Changes in birch pollen allergen-related antigen presentation upon allergen nitration revealed by MAPPs.	Mechanistic
Adamopoulou et al. (16)	Identification of peptide repertoire eluted from whole thymus, isolated thymic mDCs and mDC-depleted APCs.	Mechanistic
Scally et al. (17)	T2 cell lines transfected with HLA DRB1*04:01, 04:02, and 04:04 analyzed by MAPPs, enabling an in-depth analysis of the repertoire of bound peptides and establishment of binding motifs.	Mechanistic
Rombach-Riegraf et al. (5)	Increased mAb-derived peptide presentation upon mAb aggregation via artificial heat stress.	Mechanistic
Webster et al. (18)	Comparison of naturally presented HLA class II peptide pattern of human IgG1 allotypes G1m3 and G1m1,17, presented by allotype matched and mismatched donors.	Mechanistic
Sorde et al. (19)	MAPPs assay on IVIg-loaded DCs reveals a broad repertoire of presented IgG-derived peptides.	Mechanistic

**Figure 1:**
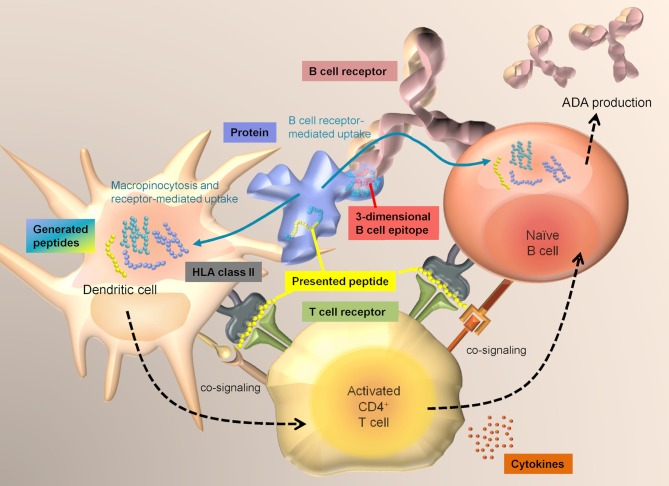
Interplay between DCs, T cells, and B cells. Dendritic cells sample antigens mainly via macropinocytosis and receptor-mediated uptake and have access to a broad range of extracellular proteins. In contrast, B cells mainly take up antigens via specific recognition of structures on an antigen via their surface B cell receptor. T cells that have become activated by specific recognition of an antigen-derived HLA class II-associated peptide on DCs via their T cell receptor, can in turn activate B cells that are presenting the same sequences.

T cell immunity is a key element in the development of immunogenicity against proteins. Specific T cell receptor-mediated recognition of HLA class II associated peptides presented by professional antigen presenting cells (APCs), as well as co-signaling via co-stimulatory molecules and cytokines are required to activate CD4+ T cells. For the development of an adaptive immune response, help by CD4+ T cells is required to mount an efficient B cell response and the production of high-affinity IgG class antibodies (Figure 1). Naturally presented HLA class II-associated peptides, which are the prerequisite for a specific T cell response, can be determined via MAPPs.

**Figure 2:**
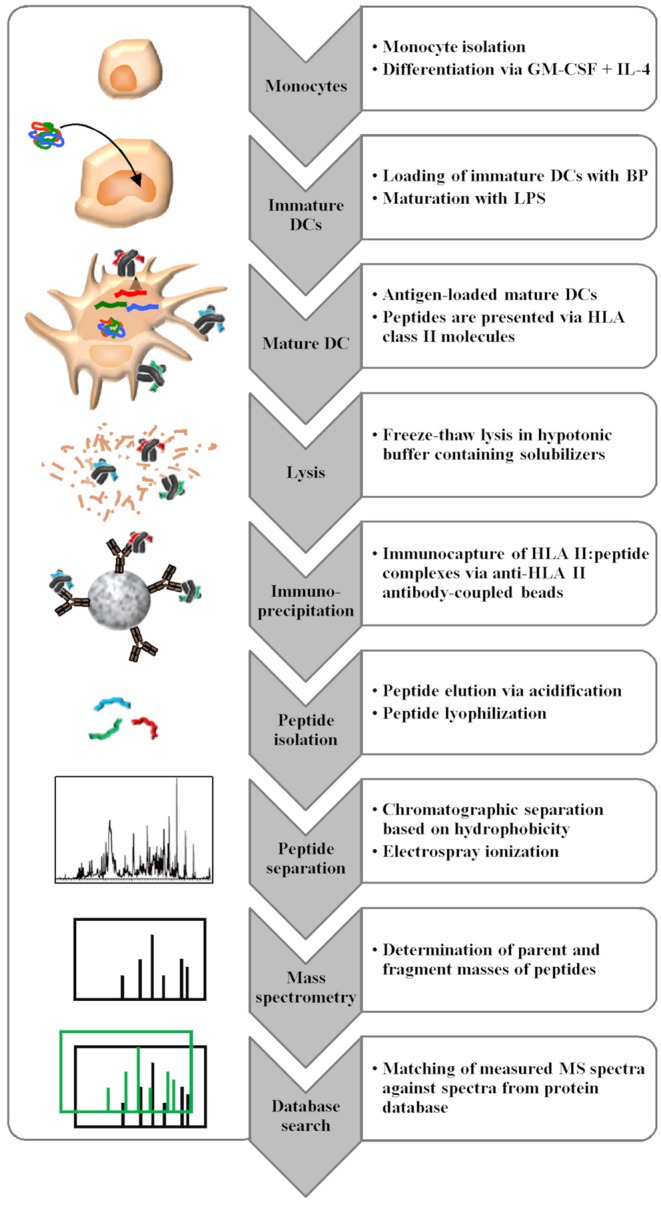
MAPPs assay procedure consisting of multiple steps. Monocytes isolated from buffy coats are differentiated *in vitro* into DCs, which are loaded with the BP of interest. After lysis, HLA class II molecules are isolated by immunocapture, and eluted peptides are analyzed via LC-MS with subsequent database search for peptide identification.

The MAPPs assay consists of multiple steps, involving human primary antigen presenting cell culture, peptide isolation, and peptide identification via liquid chromatography-mass spectrometry (LC-MS). Professional antigen presenting cells are loaded with proteins of interest, which are internalized and enzymatically processed to peptides in the endolysosomal compartment of the cells. The binding to HLA class II molecules is dependent on the properties of the amino acid side chains of a given peptide and the binding pockets of a given HLA class II molecule (2). HLA class II molecules are highly polymorphic and their peptide binding groove is open at both ends enabling the binding of different peptide length variants (3). Despite the high polymorphic diversity, the peptide binding repertoire of HLA molecules is not unlimited and there is an overlap in peptide binding specificities between HLA types (4). Following incubation with the protein of interest, the naturally processed and presented peptides are retrieved from the APCs by cell lysis, followed by membrane solubilization. Then, HLA class II: peptide complexes are isolated by immunocapture using anti-HLA II antibody-coated beads. In the following wash steps, solubilizers are removed before the peptides are eluted from the HLA class II binding groove by pH shift and are further analyzed via immunopeptidomics, which is defined as the characterization of peptide ligands bound to HLA and related molecules. Peptides are separated according to their hydrophobicity via liquid chromatography prior to electrospray ionization and mass spectrometric analysis. The determination of the full mass and fragment masses of the peptides enables sequence identification via database search by comparing the measured mass spectra to calculated mass spectra generated from peptide sequences derived from a large protein database. The identified peptides are then mapped to the sequence of the BP (Figure 2). Identified peptides often accumulate in several sequence regions of the BP, referred to as “clusters” (5, 6). Since different donors will only present certain sequence regions/clusters based on their HLA class II alleles, the analysis of about 20 donors is required to cover most of the presentable sequence regions.

## MAPPs Assay Applications

Due to the challenges with immunogenicity against biotherapeutics, methods that could potentially predict immunogenicity in human beings would be a game changer for drug development. One of the major caveats is the high variability of the published clinical immunogenicity data. Reported immunogenicity incidences are influenced by a multitude of aspects including patient-, treatment-, and sampling-related factors. Furthermore, they are impacted by the sensitivity, drug tolerance, and type of immunogenicity assay used to measure anti-drug antibody (ADA) responses in patients. A meaningful correlation between *in vitro* data and clinical immunogenicity of different marketed biotherapeutics would require the generation of clinical immunogenicity data with harmonized assays and sampling in clinical trials, which is practically utopian to achieve.

The development of immunogenicity in an individual subject is dependent on multiple factors such as the presentation of BP-derived peptides via HLA class II molecules, the recognition of these peptides as well as co-stimulatory signals by T cells, the precursor frequency of responsive T cells, the recognition of the BP by B cells via the B cell receptor, the precursor frequency of such B cells, the efficiency of cell interaction in the lymph node and resulting affinity maturation, immune status, HLA haplotype, and the target biology of the BP, just to name a few. The MAPPs assay is covering one of the key contributing factors, the natural presentation of protein-derived peptides to T cells, which is the prerequisite for the development of a specific IgG-type immune response. The ability of the peptides to trigger T cell responses has to be addressed via subsequently applied T cell assays. Since the development of immunogenicity and the incidence of ADA in the patient population are depending on many more factors, the MAPPs assay data should never be considered as a direct prediction of immunogenicity incidence in human beings. Instead, MAPPs should be understood as a useful and relevant tool to: (1) rank similar protein variants regarding their immunogenicity potential and support candidate selection, (2) identify root causes for clinical immunogenicity, (3) confirm the sequences predicted by *in silico* algorithms to characterize and further improve them, and (4) improve the mechanistic understanding of principles of antigen presentation as well as factors that are contributing to the development of immunogenicity.

### Selection of Biotherapeutic Candidates by MAPPs Assay Ranking

Due to the abovementioned caveats, absolute immunogenicity incidence rates between marketed BPs cannot be directly compared. Reported ADA incidences for a given BP can also vary significantly across studies depending on indication, co-medication, and assay format. Still, it becomes apparent that some BPs seem to have relatively low reported immunogenicity rates across many studies and indications, while other BPs seem to consistently show higher immunogenicity incidence rates. We have previously applied MAPPs and T cell activation assays on a panel of marketed monoclonal antibodies, secukinumab, adalimumab, ustekinumab, infliximab, and rituximab (6). In this study, molecules that showed on average a rather low clinical immunogenicity, also showed lower numbers of presented sequence regions and low T cell response rates. In contrast, monoclonal antibodies with elevated clinical immunogenicity rates also showed increased numbers of presented sequence regions and increased T-cell response rates in T-cell activation assays, indicating an approximate correlation between *in vitro* assay results and clinical immunogenicity incidence (6). This study indicates, that the number of presented sequence regions may be a useful information to rank similar BP candidates during drug development. Since differences in the amino acid sequences of BPs will impact on the type and number of presented peptides, the MAPPs assay should best be applied for the ranking of similar BP variants as typically generated during the candidate selection phase in drug development.

### Interrogation of Clinical Immunogenicity

The ABIRISK Project (www.abirisk.eu) of the European Innovative Medicines Initiative (IMI) investigated the correlation between patient, clinical factors and the incidence of immunogenicity and examined the underlying mechanisms of immunogenicity. As part of this project, the MAPPs technology was applied to identify naturally presented sequence regions of several biotherapeutics.

#### Infliximab

In the scope of the ABIRISK project, infliximab, a chimeric monoclonal anti-TNFα antibody, was examined via MAPPs and T cell epitope mapping assays using different sets of healthy donors as well as patients with inflammatory bowel disease or rheumatoid arthritis that had developed immunogenicity against infliximab. Despite the use of different donors, most of the identified T cell epitopes were also identified as naturally presented peptides by MAPPs. As expected, not all presented sequence regions identified in the 34 healthy donors via MAPPs were identified as true T cell epitopes in the sets of 16 healthy drug-naïve donors and 6 ADA positive treated patients. This can partially be explained by lower patient numbers and smaller HLA coverage in the T cell assay. Moreover, the T cell repertoire is shaped by central tolerance and peripheral tolerance, which are both impacting on the T cells precursor rates against different peptide sequences, and which are ultimately determining whether a donor will or will not respond. An independent study also employed MAPPs on the determination of presented sequence regions of infliximab on 9 donors (7). The comparison of both independent datasets reveals a high degree of similarity in terms of the location of presented sequence regions. In both studies, clusters in sequence regions were detected that were matching confirmed T cell epitope hotspots in patients showing immunogenicity (8), specifically: (1) a region slightly upstream of and spanning across the HCDR2 and (2) a region slightly upstream and spanning across HCDR3 and (3) a framework region in LFR1. Additional presentation hotspots were identified in both studies in HFR3 as well as in LFR3. Only one prominently presented sequence region in LCDR3 was only detected in the study by Hamze et al., likely due to the larger set of tested donors.

#### Adalimumab

Adalimumab, a human monoclonal anti-TNFα antibody, was tested in the MAPPs assay as part of the ABIRISK project (9) on 18 healthy donors. Eight clusters were identified in the VH region and four clusters in the VL region. Four of these presented regions, HCDR2, HFR3, HCDR3, and LCDR2 were also confirmed as T cell epitopes on an independent set of 14 healthy drug-naïve donors. In an unrelated study, Sekiguchi et al. evaluated the presented sequence regions on a small set of 2 donors (7). Still, three presented sequence regions could be observed in VH and three in the VL region. Almost all of these clusters matched regions identified by Meunier et al., except for 1 cluster in LFR1 that was only identified by Sekiguchi et al. This discrepancy can be explained by the use of a different anti-HLA class II clones G46-6 and L243 in the two studies. Interestingly, this cluster in LFR1 was identified in our hands using anti-pan class II antibody clone IVA12 in an independent study (data not shown).

#### Natalizumab

**Figure 3:**
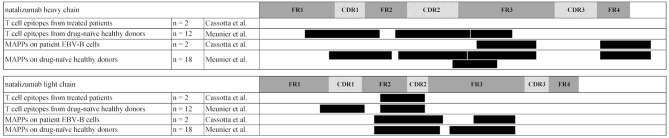
Natalizumab cluster map of presented sequence regions identified via MAPPs and T cell epitopes identified via epitope mapping. Clusters are indicated as black boxes, complementary-determining regions are indicated as shaded areas along the sequence in heavy chain and light chain, framework regions are depicted in gray. Permission to generate this figure was obtained from Dr. Luca Piccoli (10).

Two groups, Cassotta et al. and Meunier et al. applied MAPPs to identify naturally presented peptides from natalizumab, a humanized monoclonal antibody directed against α4-integrin. In the study of Meunier et al., which was performed as part of the ABIRISK project, T cell epitopes resided in HCDR1, HCDR2, and HFR3 as well as LCDR1 and at the interface between LFR2 and LCDR2 (9). Presented sequence regions identified via MAPPs were detected in the same regions, except LCDR1, likely due to the use of different donor sets. Additional presented regions resided in HFR4 and LFR3. In an independent study, Cassotta et al. applied MAPPs on natalizumab-specific EBV B cell clones that were isolated from two patients who had developed immunogenicity against natalizumab. The researchers could identify 3 naturally presented sequence regions that matched the regions identified in the ABIRISK study. Two of these regions were located in HFR3 and the HFR4, both of which did not induce specific T cell clones. The third presented region at the interface of LFR2 and CDR2 was also recognized by natalizumab-reactive T cell clones (10). This study shows, that natalizumab-specific B cells were able to recognize and internalize natalizumab via their BCR and subsequently present a natalizumab-derived peptide sequence that was verified as a natalizumab-specific T cell epitope. The interplay of linear HLA-DR associated peptide presentation by APCs, subsequent T cell response and T cell-mediated activation of B cells which are also presenting linear HLA-DR associated peptides upon BCR mediated antigen-specific protein uptake (Figure 1) are demonstrated in this study (10). A comparison of presented sequence regions and identified T cell epitopes is shown in Figure 3.

#### Ixekizumab

In a recent study (11), Spindeldreher et al. identified T cell epitopes of ixekizumab, a chimeric monoclonal antibody directed against IL-17a, from 31 healthy treatment-naïve donors via MAPPs-assisted T cell epitope mapping. All identified T cell epitopes resided in four main regions of the mAb, which were overlapping with the regions HCDR3, LCDR1, LCDR2, and LCDR3. For the epitope mapping approach, synthesized peptides from overlapping peptide libraries were used as well as peptides synthesized from presented sequences identified via MAPPs. We concluded, that the approach of MAPPs-assisted T cell epitope mapping appeared to be superior over “traditional” peptide-scanning approaches, and that the sequences identified by MAPPs represent antigenic regions which underwent intracellular processing of the full-length protein and may thus present “authentic” T cell epitopes.

#### Human Factor VIII

**Figure 4:**
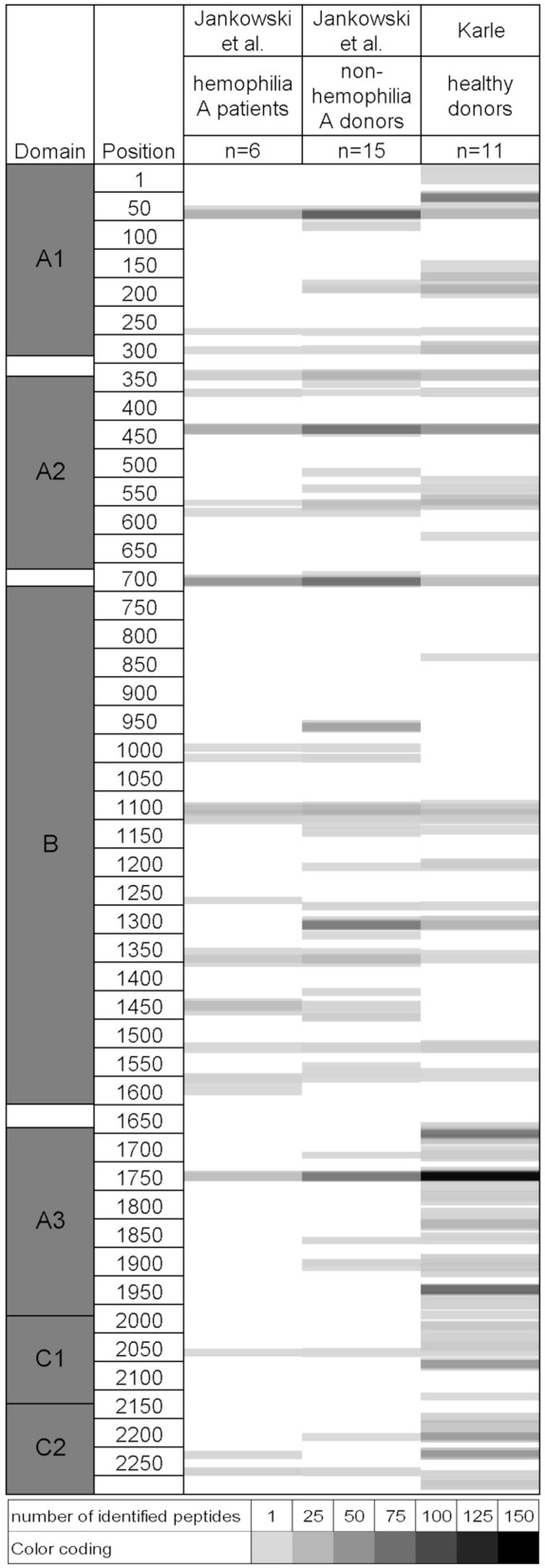
Comparison of naturally presented sequence regions in two independent studies on human Factor VIII identified via MAPPs. Left: Factor VIII protein domains and amino acid positions. For each dataset, identified peptides are indicated as shaded areas along the amino acid sequence from top to bottom. The number of identified peptides per position is indicated by grayscale color coding. Raw data and permission to generate this figure was obtained from Dr. Zuben Sauna, Joseph McGill, and Dr. Wojciech Jankowski (12).

The immune response to factor VIII is a significant complication in the treatment of patients with hemophilia A. In a recent study, MAPPs was applied to identify potential T cell epitopes of human factor VIII (12). A similar dataset was generated in our group in the scope of the ABIRISK project and a list of identified factor VIII-derived peptides is available in Supplementary Data 1. A comparison of the two datasets reveals a considerable similarity in presented sequence regions despite the use of completely independent healthy donor sets with potentially different HLA allele representation, as shown in Figure 4. Differences between the studies are observable in domains B, A3, C1, and C2. In the B domain, few presented regions were only identified by Jankowski et al., likely due to the higher number of tested donors. Additional sequence regions were identified in the ABIRISK study in domains A3, C1, and C2. Discrepancies between the 2 studies in the ability to detect certain sequences may be related to the HLA distribution and number of tested donors, type of tested factor VIII material and assay sensitivity.

### Improving Mechanistic Understanding of Principles of Antigen Presentation

MAPPs is a powerful tool to interrogate the mechanistic principles of antigen presentation. We have previously generated data comparing the peptide presentation between monocytes, monocyte-derived DCs (moDCs), and B cells (20). Monocytes, B cells, and moDCs presented synthesized peptides to a comparable level, which can be explained by surface exchange. In contrast, moDCs were the most efficient cells in peptide presentation from full proteins such as ovalbumin, tetanus toxoid, and trastuzumab, likely due to higher uptake efficiency via macropinocytosis. In order to compare the pattern of presented peptides, two molecules were selected that were expressed by all three cell types and which were less influenced by the differences in antigen uptake processes between cell types. For the two selected intrinsic molecules (HLA-DRA chain and HLA-B), B cells and monocytes showed the same presentation pattern. MoDCs showed additional clusters, which could be due to a higher HLA-DR expression on moDCs combined with the stronger membrane uptake via micropinocytosis, increasing the amount of presentable HLA-DRA and HLA-B derived peptides and boosting detection efficiency via MS. Alternatively, the additional clusters observed in moDCs may also be explained by differences in the composition of lysosomal proteases (21).

In another study focusing on the naturally presented peptide repertoire of APCs, Adamopoulou et al. applied the MAPPs technology to interrogate the HLA class I and class II human thymic peptide repertoire. They identified peptides eluted from whole thymus, from isolated thymic myeloid DCs (mDCs) and from mDC-depleted APCs containing pDCs, thymic epithelial cells, and other thymic APC (16). According to the authors, only about a third of the protein pool was shared between mDCs and non-mDC cells and the authors concluded that the differences in the presented peptide repertoire are supporting the idea that different thymic APCs present different peptides. The identified HLA class II peptide sequences provided in Supplementary Data 1 of that study reveals, that within each dataset, several peptide families/clusters with shared core sequences were identified, each comprising several similar peptide lengths variants. Therefore, a comparison of peptide clusters rather than distinct peptide sequences is more meaningful, and shows that a presentation similarity between the mDC and mDC-depleted samples is actually much higher than postulated in the publication.

Another mechanistic study employed MAPPs to interrogate two potential modes of action of intravenous immunoglobulin (IVIg), which is commonly used in the clinic to treat autoimmune and severe inflammatory diseases (19). Numerous IgG-derived peptides were presented by moDCs along the antibody sequences, while surprisingly regulatory T cell epitopes 167 and 289 were not efficiently presented. Some sequences cannot be efficiently identified due to technical reasons such as strong hydrophobicity preventing efficient elution from the chromatography column, poor ionization and fragmentation in the mass spectrometer, or degradation of the peptides due to extensive protease activity during cell culture. To rule out that regulatory T cell epitopes 167 and 289 were potentially missed in the experiment due to technical challenges, the researchers confirmed efficient detection via mass spectrometry by direct infusion. They could also show that in DCs loaded with synthesized regulatory T cell epitopes 167 and 289, these sequences were efficiently presented and identified via MAPPs. The results suggest that regulatory T cell epitopes 167 and 289 can be efficiently presented by DCs and detected via MAPPs but only when provided to the cells as synthesized peptides, whereas they do not seem to be efficiently processed and presented as part of a full antibody protein. In another experiment the researchers could show that the simultaneous loading of moDCs with Ovalbumin and high concentrations of IVIg did not impair Ovalbumin-derived peptide presentation, indicating that a sufficient amount of empty HLA class II molecules are present in the endolysosome to bind all generated peptides. The absence of antigenic competition was confirmed by analyzing peptides derived from two endogenous self-proteins that are typically presented on HLA class II molecules, ANXA2 and MAN2B1, which also remained unaffected by co-loading of the cells with high doses of IVIg. The authors concluded that the clinical effects mediated by IVIg are neither caused by Tregitopes 167 and 289 nor by impaired antigen presentation.

Webster et al. investigated the potential of human IgG1 allotypes to stimulate CD4+ T cell responses in donors matched for homologous and heterologous IgG1 allotypes (18). No significant responses against allotypic variants of trastuzumab were observed in allotypic mismatched T cells. MAPPs was employed to investigate the lack of T-cell responses in relation to mismatched allotypes and revealed that no peptides from the sequence regions containing the allotypic variations were presented, and thus, no T cell response was to be expected. The authors concluded that allotypic differences in human IgG1 do not represent a significant risk for induction of immunogenicity.

### Assessing the Impact of PTMs, Folding, and Aggregation on Antigen Presentation

Studies have shown that post-translational modifications (PTMs) or aggregation can affect peptide presentation by APCs (5, 15). Using the MAPPs assay, nitration of birch pollen allergen occurring in polluted air has been shown to result in enhanced presentation of allergen-derived HLA-DR-associated peptides. Both, the copy number of allergen-derived peptides as well as the number of presented sequence regions were increased for the nitrated allergen, likely due to changed processing in the endolysosome (15). Frequently recognized T cell–activating regions and peptides created by endolysosomal allergen degradation identified in a related study (22), corresponded to the birch pollen allergen derived peptides identified via MAPPs (15).

In another study we demonstrated that aggregation plays an important role in antigen presentation. Highly aggregated solutions of two model mAbs generated under exaggerated stress conditions induced strong changes in the pattern and quantity of mAb-derived HLA-DR associated peptides presented by DCs (5). An apparent linear relationship was established between the amount of protein contained within the subvisible particles and the increase in presentation. We postulated that the internalization of large densely compacted proteinaceous particles would rapidly increase the effective protein concentration in the endolysosome as compared to uptake of soluble monomeric material and thereby boost presentation.

Jankowski et al. have shown that folding, post-translational modifications or glycosylation patterns of different tested FVIII preparations may contribute to differential enzymatic processing and presentation (12). Plasma-derived FVIII showed fewer presented peptides compared to full length recombinant FVIII.

Since the MAPPs assay has been shown to be sensitive for aggregation, PTMs and alterations in protein folding, the assay can serve as a useful tool to further interrogate aggregation- and protein-structure-related mechanisms of immunogenicity.

## Advantages and Challenges of MAPPs

The major advantage of MAPPs is the identification of relevant peptides that have been naturally processed and presented by primary professional APCs. On the other hand, MAPPs is a costly and time consuming assay and requires cutting edge instrumentation as well as a significant level of expertise to be able to generate high quality data. In our group, hands-on time and turnaround time could be reduced by (1) introducing automation during cell isolation via a robotic system, (2) implementation of a robotic system for bead-washing during peptide isolation, (3) automated LC-MS sample loading and dual column-switching in front of the mass spectrometer to increase throughput, (4) implementation of tailored in-house data processing softwares to significantly reduce the time required for data analysis and reporting. Still, turnaround times for the ranking of up to 10 BP candidates on 24 donors in the MAPPs assay are in a range of 8 weeks, which can sometimes pose a challenge to fit into the candidate selection phase. More importantly, the material amounts and purity required for the MAPPs assay can be a challenge during early phases of a project when BPs are produced in small scale.

### MAPPs vs. *in silico* Algorithms

*In silico* approaches are often considered an alternative approach to MAPPs due to the short turnaround time, low resource requirements and the fact that no BP material is required. Recently, tools have been developed to predict hot spots at protein-protein interfaces (23, 24), but still most of the available tools are classical algorithms based on HLA class II binding data. Although these methods are more cost effective, less time consuming, and easier to perform, the MAPPs assay provides more meaningful and relevant information. The advantages and limitations of these *in silico* tools for the identification of HLA class II binders have been discussed elsewhere in detail (25). Briefly, most algorithms are trained on data from HLA class II peptide binding experiments which are partially biased by selection, length and solubility of test peptides as well as the folding of the synthetic HLA molecules. Therefore, only a small fraction of the naturally occurring peptidome is represented in the training dataset. Moreover, resulting algorithms are typically not considering the binding contribution of flanking regions outside of the 9-mer binding core.

In contrast, the MAPPs assay enables the identification of relevant peptides that have been naturally processed and presented by primary professional APCs. These cells contain a complex mixture of enzymes that shape the peptide repertoire. While some generated peptides may be able to bind to HLA class II molecules, other sequences may be degraded and removed from the repertoire of presentable peptides.

MAPPs data also offers the opportunity to train new HLA class II binding prediction algorithms, with the potential to improve the predictive power of such new computational tools (manuscript in preparation). Moreover, MAPPs data generated on a multitude of antibodies may help to identify presented sequence stretches that are conserved across antibodies, and which are unlikely to contribute to the immunogenic potential of a given antibody.

### MAPPs vs. HLA: Peptide Binding Assays

A previous study by Hamze et al. compared T cell epitope mapping data, MAPPs data, and HLA binding assay data (8). HLA-DR binders identified for the monoclonal antibodies rituximab and infliximab covered most of the variable antibody sequence, which did not allow for proper matching with T cell epitope data. In contrast, presented sequence regions identified via MAPPs were restricted to several distinct areas of the two molecules, which also excellently aligned with T cell epitopes identified from drug-naïve healthy donors as well as treated patients who had developed immunogenicity against rituximab or infliximab (8). While peptides with a fixed length or with poor solubility may give rise to false negative results in HLA-peptide binding assays, they may still be detected via MAPPs, since partially unfolded protein fragments can bind to HLA class II molecules with subsequent trimming by enzymes according to the “bind first, trim later” model (26). Moreover, the chaperone HLA-DM promotes the formation of stable HLA class II peptide complexes (27). This peptide repertoire-editing functionality of HLA-DM is not reflected in binding assays, but it is an integral part of the natural peptide processing in the cells used for the MAPPs assay.

### MAPPs vs. T Cell Assays

APCs present a variety of potential T cell epitopes on their surface, and specific reactive T cells in the blood of an individual are required to induce a T cell response. As a result, not every presented sequence region in an individual subject is necessarily acting as a T cell epitope. The MAPPs assay is unable to discriminate between sequences that will or will not induce a T cell response. T cell assays are often employed using full proteins, and the percentage of responders in a cohort of healthy drug-naïve donors is determined. While such assays yield a response rate, they are not useful in identifying the epitopes that triggered the immune response. Peptide-based T cell assays are suitable to identify T cell epitopes but epitopes with low specific T cell precursor frequencies may be missed. Also, T cell proliferation against immune-dominant epitopes will overgrow responses against weaker epitopes, so that the true number of T cell epitopes in a molecule can be underestimated. Ideally, MAPPs and T cell epitope mapping assays are used in conjunction: Presented sequences should be identified via MAPPs and the identified peptides of interest subsequently synthesized for down-stream T cell epitope mapping. An example of such a combined approach is detailed in the section on MAPPs assay applications in the paragraph on ixekizumab.

### Impact of Assay Sensitivity on Assay Application

The sensitivity of the MAPPs assay is a crucial factor that requires adequate tuning for the different areas of application. While de-immunization approaches require a high sensitivity that enables the detection of all presented sequence regions within a molecule, ranking approaches should rather be tuned for a large dynamic range. A minimum number of copies of a given peptide is required to generate a decent fragment spectrum that will enable confident sequence identification via databases search. While too low amounts of available peptides will not consistently yield spectra qualities allowing for peptide identification, high amounts of injected peptides will lead to signal saturation resulting in plateauing peptide identifications due to speed limitations of the mass spectrometer. Signal saturation is advantageous for de-immunization approaches when all presented sequence regions need to be detected to be able to identify the sequences that should be de-immunized. For the ranking of different biotherapeutics, the differences in the amount of presented peptides are of relevance and thus, signal saturation should be avoided. In conclusion, MAPPs assays should be carefully tuned to the purpose rather than using one standard approach for all applications. The assay sensitivity is based on several key parameters: (1) Cell culture conditions: The amount of BP-derived peptides available for analysis is dependent on the differentiation and maturation status of the cell, the amount of expressed HLA class II molecules per cell, the number of cells used for peptide isolation, and the BP-concentration in the cell culture, the latter of which determines how readily the BP is taken up and processed by the APCs. (2) Peptide isolation methods: Different peptide isolation methods have been published using various detergents for membrane solubilization as well as different washing techniques to remove detergent and unrelated proteins after immunoprecipitation (5, 6, 28). All of these aspects can impact on the amount and purity of peptides that can be retrieved for analysis and on potential signal quenching by residual detergent in the peptide preparation as well as leachates from plastic surfaces. (3) Chromatography: An efficient and robust chromatographic separation prior to MS analysis is required for the identification of naturally presented HLA class II peptides. The column chemistry, use or non-use of a pre-concentration column, applied solvents, gradient profile, flow rate, column length, as well as the amount of peptides loaded onto the column will impact on peak separation, peak sharpness, signal intensity and ultimately on peptide identification. (4) Mass spectrometry: The challenges of immunopeptidomics, have been reviewed in detail by Faridi et al. (29). For MAPPs analyses, mass spectrometers with high-mass-accuracy are commonly applied (5–7, 28, 30). Measurement speed and mass accuracy are key parameters that enable identification of peptides in the highly complex HLA class II peptide samples. Often, a dynamic exclusion list is applied (30), which is preventing previously measured high intensity peaks from being triggered continuously, and which is enabling the detection of low abundant peaks. (5) For peptide identification based on the measured mass spectra, database search algorithms such as SEQUEST and MASCOT are commonly employed (5–7, 30). Settings are typically adjusted to limit false positive identification rates to a maximum of 1–2% (28, 30). One major challenge of the database search approach is that peptides with PTMs of unknown mass or glycosylated peptides will not be identified, as the measured masses will not match the expected masses.

## Summary and Conclusion

Despite the fact that a clinical validation of the MAPPs assay will remain a challenge, a biological validation has been shown in several cases over the past years. Data generated as part of the ABIRISK project as well as independent studies show that presented sequence regions identified via MAPPs matched T cell epitopes identified from drug naive healthy donors and treated patients that had developed immunogenicity (7–12). Together, these studies show that MAPPs assays applied by independent research groups and performed on different donor sets yield comparable results. Sequences identified by MAPPs were shown to be meaningful and biologically relevant for the T cell responses observed clinically and can therefore help to understand immunogenicity against BPs. MAPPs data has also been shown to be more meaningful than soluble HLA class II peptide binding assay data (8). MAPPs assays have been shown to be a useful tool to interrogate clinical immunogenicity root causes by determining the natural presentation of peptides and confirming the relevance of T cell epitopes that have been identified via peptide library T cell epitope mapping approaches (7–11). Moreover, recent data suggests, that MAPPs-assisted T cell epitope mapping with MAPPs assay-derived peptide sequences could enable the identification of “authentic” T cell epitopes (11). Also, databases that compile previously identified T cell epitopes could be useful tools to be applied in conjunction with MAPPs. MAPPs has successfully been applied in a range of mechanistic studies, to interrogate the effect of PTMs (15), folding (12), and aggregation on antigen presentation (5). The technology has also been proven valuable for comparing the antigen presentation across different APC cell types (16, 20), for determining naturally presented sequence regions of IVIg (19), and for evaluating the potential of human IgG1 allotypes to stimulate T cell responses in donors matched for homologous and heterologous IgG1 allotypes (18). As the MAPPs assay has shown the capacity to differentiate molecules regarding their content of potential T cell epitopes (6), the technology can also be useful to support candidate ranking and selection during early drug design and may contribute to the development of BPs with lower immunogenicity.

## Materials and Methods

Determination of Factor VIII-derived naturally presented HLA class II-associated peptides as part of ABIRISK project: Naturally presented HLA class II–associated peptides were identified via the MAPPs assay (6) from 11 healthy donors' monocyte-derived DCs exposed to recombinant human Factor VIII (Octocog alpha), kindly provided by Dr. Dr. med. Christoph Königs from Universitästsklinikum Frankfurt. CD14 positive mononuclear cells were purified from PBMC collected from consented healthy donors (Blood Donation Center Bern, Bern, Switzerland) and differentiated into immature DCs. Immature DCs were loaded with human Factor VIII at a final concentration of 0.6 nmol/ml in the cell culture, matured with LPS (1 μg/mL, Sigma) and incubated for 24 h at 37°C and 5% CO_2_. HLA class II molecules were immunoprecipitated with anti-HLA-DR/DP/DQ monoclonal antibody IVA12-conjugated beads and peptides were eluted from HLA class II molecules by adding 0.1% trifluoroacetic acid (Fluka, Buchs, Switzerland) at 37°C. Lyophilized peptides were re-suspended in hydrophilic buffer containing 5% acetonitrile and 1.1% formic acid. Peptide composition was analyzed by liquid chromatography (nano capillary system, Dionex Corporation, Sunnyvale, California, USA) on a self-packed fused-silica C18 reversed-phase nano-high-performance liquid chromatography column connected to a mass spectrometer (Q-Exactive HF Biopharma, Thermo, California, USA) via electrospray ionization (LC-ESI-MS/MS). Peptides were identified via a database search approach using the SEQUEST algorithm as detailed previously (6).

## Author Contributions

AK wrote all sections of the manuscript.

## Conflict of Interest

AK is an employee in, and has stocks and stock options at Novartis.

## Supplementary Material

Supplementary Data 1 Complete list of Factor VIII-derived peptides per donor identified in the scope of the ABIRISK project.

## Abbreviations

PK/PD: pharmacokinetics/pharmacodynamics
mAb: monoclonal antibody
DC: dendritic cell
APC: antigen presenting cell
BP: biotherapeutic protein
HLA: human leukocyte antigen
mDC: myeloid dendritic cell
moDC: monocyte-derived dendritic cell
pDC: plasmacytoid dendritic cell
ADA: anti-drug antibodies
MAPPs: MHC associated peptide proteomics
PTM: post-translational modification
MS: mass spectrometry
LC: liquid chromatography
CDR: complementary determining region
FR: framework region
HCDR: heavy chain complementary determining region
LCDR: light chain complementary determining region
HFR: heavy chain framework region
LFR: light chain framework region.
